# Evaluation of Mechanical Performances of Stents with 38 mm Length in Long Lesion

**DOI:** 10.1155/2020/2594161

**Published:** 2020-02-27

**Authors:** Xiaoting Yue, Jiacheng Guo, Jianchao Zhang, Chang Cao, Zenglei Zhang, Deliang Shen, Junnan Tang, Jinying Zhang

**Affiliations:** ^1^Department of Cardiology, The First Affiliated Hospital of Zhengzhou University, Zhengzhou, Henan 450052, China; ^2^Henan Province Key Laboratory of Cardiac Injury and Repair, Zhengzhou, Henan 450052, China

## Abstract

**Objective:**

Single stent with 38 mm length has emerged as a potential solution for long lesion treatment using PCI. However, long stents need to come over a longer lesion length combined with a higher incidence of tortuous calcification, requiring a stent to provide superior transport and compliance and reduce elastic retraction. Here, we evaluated the mechanical performances of the existing four types of drug eluting stents with 38 mm length, which could provide guidance for clinicians to choose the proper stents for the patients.

**Methods:**

The stents with 38 mm length from XIENCE Xpedition (Abbott, US), SYNERGY (Boston Scientific, US), FIREHAWK (Microport, China), and HELIOS (HELIOS, China) were collected. Mechanical parameters of stents including crossing ability, compliance, elastic recoil, and longitudinal strength were performed.

**Results:**

The resistance force of stents from XIENCE Xpedition was smaller than FIREHAWK (*p* < 0.05), which indicates that the stent from XIENCE Xpedition has better crossing ability. The ratio of stent diameter reduction from both XIENCE Xpedition and SYNERGY was less than 3% with no statistical difference. In addition, the elastic recoil percentage of stents from SYNERGY, XIENCE Xpedition, FIREHAWK, and HELIOS was 1.16%, 2.62%, 3.66%, and 4.19%, respectively, indicating that SYNERGY had better elastic recoil compared to FIREHAWK and HELIOS (*p* < 0.05), which indicates that the stent from XIENCE Xpedition has better crossing ability. The ratio of stent diameter reduction from both XIENCE Xpedition and SYNERGY was less than 3% with no statistical difference. In addition, the elastic recoil percentage of stents from SYNERGY, XIENCE Xpedition, FIREHAWK, and HELIOS was 1.16%, 2.62%, 3.66%, and 4.19%, respectively, indicating that SYNERGY had better elastic recoil compared to FIREHAWK and HELIOS (*p* < 0.05), which indicates that the stent from XIENCE Xpedition has better crossing ability. The ratio of stent diameter reduction from both XIENCE Xpedition and SYNERGY was less than 3% with no statistical difference. In addition, the elastic recoil percentage of stents from SYNERGY, XIENCE Xpedition, FIREHAWK, and HELIOS was 1.16%, 2.62%, 3.66%, and 4.19%, respectively, indicating that SYNERGY had better elastic recoil compared to FIREHAWK and HELIOS (

**Conclusion:**

The evaluation of mechanical properties for the stent with 38 mm length including crossing ability, compliance, elastic recoil, and longitudinal strength could provide reference index for more accurately clinical application for long lesion treatment.

## 1. Introduction

Complex coronary lesions, especially the long lesion, still remain a challenge for intervention cardiologists, which usually require multiple stent implantations. The coronary artery lesion greater than 20 mm is defined as a long lesion. Percutaneous coronary intervention (PCI) preformation in long lesion could be the overlapping of several stents, and in fact, stent overlap has been reported in as many as 30% of patients undergoing PCI. In the age of bare metal stents (BMS), stent length was an independent predictor of in-stent restenosis (ISR) [1]. Thus, often, PCI operation was not recommended for the long lesion.

With the development of the drug-eluting stent (DES), after the year of 2000, the risk of ISR dropped from 20%–30% in the BMS era to 5%–10% in the DES era [2–6]. However, the late restenosis rate after DES implantation is still relatively high [7], especially multiple stent implantation. The common approach for current clinical setting still involves the overlapping of two short stents. Unfortunately, the overlapping method in clinical application is deficiency in some extent. Multiple stent implantation could result in the excessive overlap of double-layer stent beams or the geographical loss between the overlapped two short stents easily, together with the prolonged operation time and extensive medical expenses. For some type of long lesions, the overlapping length of stents can be either too long or too short. While both of excessive overlap and longer total length of the stents by less overlapping may lead to an excessive incidence of ISR [8–10]. In addition, the medical cost for cardiac patients could increase because of the usage of multiple stents [11]. Previous reports also indicated that stent overlap was associated with major adverse cardiac events (MACE) [2, 12, 13].

However, recent clinical reports have shown that overlapping latest generation of DES is safer and more efficient, compared to early-generation DES [12, 14]. Moreover, new stent designs with increasing length are emerging as an alternative tool to reduce the needs of overlapping multiple stents, during the percutaneous treatment of coronary artery disease (CAD). Therefore, to treat the long lesion during PCI became feasible [13, 15, 16]. In fact, one single newer-generation DES with 38 mm in length has been applied into clinical use for some specific patients. From a clinical and economic perspective, the utilization of single stents can significantly reduce the practice of overlapping stents, resulting in lower ISR, shorter operation time, and less cost. However, for long lesion treatment, single stents need to provide superior transport and compliance and a reduced elastic retraction, as well as an excellent longitudinal strength, which are all relevant to their design and mechanical properties [14, 17, 18].

Until now, there is no sufficient data on the mechanical properties of long stents applied for a long lesion in former studies. Therefore, the goal of the present work was to compare the mechanical performances of the existing DES with 38 mm length, which gives the cardiologists a better understanding of the performance of the current market available DES. Here, we evaluated the crossing ability, compliance, elastic recoil, and longitudinal strength of the four popular stents with 38 mm length being used in current clinical setting.

## 2. Materials and Methods

### 2.1. Samples

The stents with 38 mm length from XIENCE Xpedition (X-stent) (Abbott, US), SYNERGY (S-stent) (Boston Scientific, US), FIREHAWK (F-stent) (Microport, China) and HELIOS (H-stent) (HELIOS, China) were collected. The detailed parameters of the collected stents are presented in Table 1. The size of four stents was 3.0 ∗ 38 mm, which was defined as a long stent. The X-stent, F-stent, and H-stents were made by cobalt-chromium alloy (CoCr), and the metal material of S-stent was platinum-chromium alloy (PtCr).

### 2.2. The Method to Test the Crossing Ability

The test of crossing ability is based on the standard of YY/T 0663.2-2016, which is from the People's Republic of China Pharmaceutical Industry Standard. We used the push tester (resolution 0.001 mN) and plane vessel model with three curves as test tools. We advanced the stent catheter system through three curves (bending radius from proximal to distal: 16 mm, 12 mm, and 9 mm) in 15 mm/s speed and pulled back to the initial. The resistance force was measured, and the maximal value was used to evaluate the crossing ability.

### 2.3. The Method of the Compliance Test

The test of compliance met with the standard of YY/T 0663.2-2016. Bending test mold (cylindrical gauge) and image measuring instrument were used as the testing tools. For the compliance of stent, the stent was expanded with NP (nominal pressure, stent diameter D1 was measured) and bended along a 16 mm diameter cylinder (stent diameter D2 was measured). The reduced stent diameter %= (D1-D2)/D1 ∗ 100% could indicate the compliance of the stent.

### 2.4. The Test of Elastic Recoil

In this trial, same standard as the compliance test standard was employed. Using a vernier caliper, we measured the stent diameter (ER-D1) when the stent was expanded with NP and held the pump pressure. We then measured the stent diameter (ER-D2) when the pump was released. ER-D1 and ER-D2 was defined as the average of detected diameters in the proximal, middle, and distal location of the stent, which were measured at a different state. The elastic recoil %= (ER-D1-ER-D2)/ER-D1 ∗ 100%.

### 2.5. The Test for Longitudinal Strength

For longitudinal strength, the stent was expanded with NP and compressed by using a machine (Instron5943) with 0.1 mm/s speed from 0 N up to 0.49 N force, and the maximal compressed stent length was measured. The less compressed stent length indicated better longitudinal strength.

### 2.6. Statistic Analysis

Quantitative variables that follow a normal distribution were expressed as mean ± standard deviation or abnormal distribution expressed as median (range). Qualitative variables were analyzed as statistical significance level of *p* value less than 0.05. Comparisons among more than three groups were performed with One-way ANOVA or nonparametric analysis followed with multiple comparisons test. All statistical analyzes were performed using Graph Prism7.0.

## 3. Results

**Figure 1:**
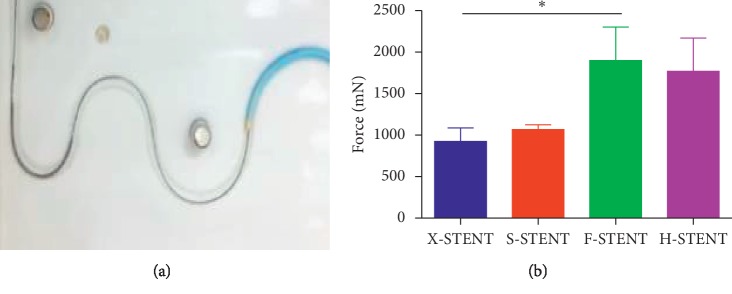
The resistance force of the stents in the test of crossing ability. The crossing ability test of the stents was completed by using a catheter system (a). In this test, the resistance force was compared among stents from the X-stent, S-stent, F-stent, and H-stent (b). ^*∗*^*p* < 0.05.

The detailed parameters of the enrolled stents were presented in Table 1. The size of four kinds of stents was 3.0 ∗ 38 mm, which was defined as one kind of long stents. In the crossing ability test, when the different stents were pushed and pulled back in 15 mm/s speed (Figure 1(a)), the average resistance force was recorded. In general, a lower resistant force indicates a better crossing ability. In this test, the average resistant force was 923.23 mN, 1066.66 mN, 1900.00 mN, and 1766.66 mN for the X-stent, S-stent, F-stent, and H-stent, respectively. When we compared the average resistance force among the four groups, it clearly showed that the F-stent generated highest resistance force, and there was a significant difference compared with this from X-stent groups (*p* < 0.05) (Figure 1(b)). Furthermore, the resistant force among the stents from XIENCE Xpedition, SYNERGY, and HELIOS was similar, which indicated better crossing ability in these three groups.

**Figure 2:**
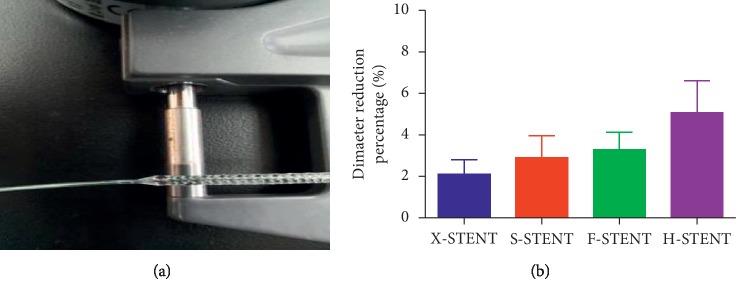
The percentage of stent diameter reduction of the stents in the compliance test. Diameters of the stents were measured both before and after the stent was bended along a 16 mm diameter cylinder (a). The reduced stent diameter% was compared among stents from the X-stent, S-stent, F-stent, and H-stent (b).

In Figure 2(a), we presented the curved stent in the standard angle for testing the compliance. The ratio of reduced stent diameter reduction is relevant to the ability of the compliance. Often smaller ration implies better compliance. In this test, the average ratio of stent diameter reduction in the X-stent, S-stent, F-stent, or H-stent stents was 2.11%, 2.89%, 3.30%, and 5.06%, respectively. According to the data, it seems that X-stents hold a lower ratio of stent diameter reduction. However, there was no statistical difference when we compared the ratio of stent diameter reduction among the four groups (Figure 2(b)).

**Figure 3:**
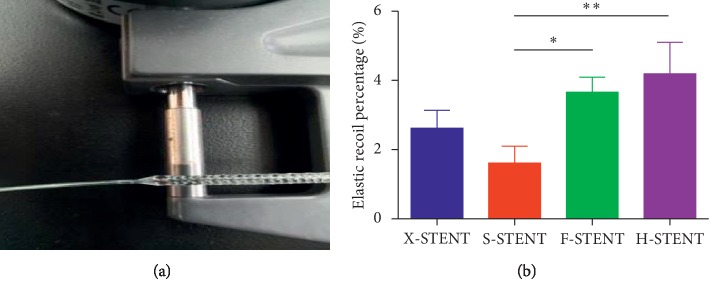
The percentage of elastic recoil of the stents. Using a vernier caliper, the stent diameter was measured before and after the pump releasing (a). The elastic recoil% was compared among stents from the X-stent, S-stent, F-stent, and H-stent (b). ^*∗*^*p* < 0.05; ^*∗∗*^*p* < 0.005.

In addition, a relatively smaller elastic recoil percentage is corresponding to an excellent stent performance (Figure 3(a)). The percentage of elastic recoil of stents from the X-stent, S-stent, F-stent, or H-stent was 2.62%, 1.16%, 3.66%, and 4.19%, respectively. Further statistical analysis indicated that the percentage of elastic recoil of stents from the S-stent was significantly lower than that from the F-stent and H-stent (Figure 3(b)). However, there was no difference between the X-stent and S-stent (Figure 3(b)), which indicates comparable performance between these two kinds of stents.

**Figure 4:**
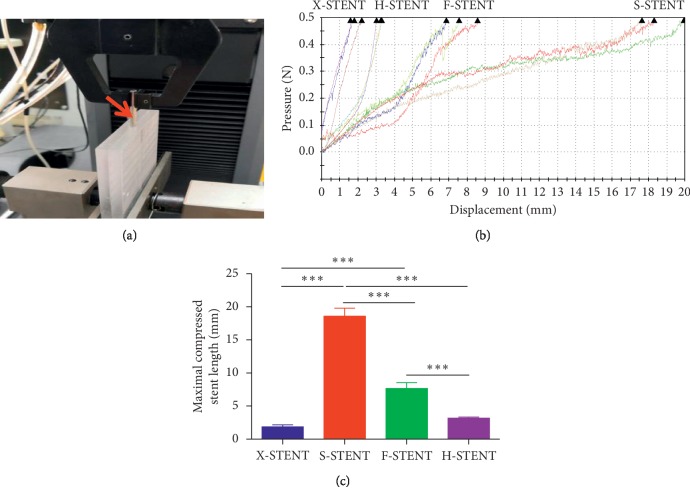
The maximal compressed stent length of the stents in the longitudinal strength test. Model 5943 Materials Testing was used in the tested of longitudinal strength (a), and the compressed stent length from different source were measured under pressure (b). The maximal compressed stent length was compared among stents from the X-stent, S-stent, F-stent, and H-stent (c). ^*∗∗∗*^*p* < 0.001.

Notably, longitudinal strength is an important indicator for the mechanical performances of DES. After being compressed by Instron 5934 and expanded by NP, the average maximal compressed stent length of X-stent, S-stent, F-stent, or H-stent was 1.86 mm, 18.6 mm, 7.65 mm, and 3.18 mm, respectively (Figure 4(a) and 4(b)). Obviously, the stents from the X-stent and H-stent had less displacement under pressure compared with the S-stent and F-stent. This result demonstrated that both X-stent and H-stent had superb longitudinal strength (*p* < 0.0001) (Figure 4(c)). Surprisingly, S-stents presented the most displacement. (Figure 4(c)).

## 4. Discussion

It has been investigated that high incidence of major adverse cardiovascular events is associated with lesion length. Coronary stenting is a milestone in the treatment of CAD [19]. However, due to the complicated properties of the long lesions, including distortion, calcification, and angle formation, it has high demand in the surgeon's skill and the overall performance of the stent. Many animal and human experiments have showcased that materials and design of stents can have an important impact on postoperative arterial intimal thickening, which is related to the formation of in-stent restenosis [20–23]. Therefore, focusing on the mechanical properties of the stent is important for reducing adverse events after PCI. With the development of stents design, the newly adopted a stent with 38 mm may potentially solve many problems in treating long lesions. However, in the current market, a variety of different 38 mm stents bring doctors some troubles in their choices. Hence, this study provides a much-needed guidance for cardiologists to choose the best stents. Here, we focused on assessing the mechanical properties of the DES with 38 mm length available in the market, to assist clinicians select the proper stents for a long lesion, which can reduce adverse events after PCI.

Due to its complexity, the long lesion is often associated with the vessel calcification, vascular circuity, and repeated operation on the stents. Especially, the application of balloons in the procedure of PCI is frequent for long lesion treatment. Better longitudinal strength of stents could prevent the stent deformation during the operated process. In our present study, mechanical parameters like crossing ability, compliance, elastic recoil, and longitudinal strength have been evaluated for those four long stents with 38 mm length, which may provide some reference for clinicians when choosing the stents for application in patients. In this study, X-stent performed significantly better than F-stent in the test of crossing ability. In terms of compliance, X-stent was better than other DES, although there was no significant difference. Thus, X-stent could be listed as one of the top choices when the long lesion companied with severe vessel calcification and vascular circuity. S-stent showed significantly improved percentage of elastic recoil when compared to F-stent and H-stent, but was no significant compared with X-stent. As to the longitudinal strength test, stents from the X-stent achieved an overwhelming advantage compared with the other three, which indicated that X-stent could be the best choice when the PCI operation needs multiple application of balloon. Inevitably, there are still some limitations in this study. The sample size of this study is relatively limited. Also, instead of assessing all mechanical parameters of DES, only important ones were tested and compared. Moreover, the assessment in this study was only performed *in vitro*, without verification from *in vivo* or human trials. Thus, further larger-scale studies with more comprehensive data are essential to provide a better insight for clinicians.

## 5. Conclusion

Mechanical performances of stent should be considered as one of the major factors when clinicians perform the operation of stent. Our present study compared the performance of the four major 38 mm long stents, which may provide a direct index for accurately clinical application. Clinicians could make more accurate decision on the choice of stent as well as the following treatment when combining the detailed mechanical performance of stent and the patient's condition.

## Figures and Tables

**Table 1:** The detailed information of the stents with 38 mm length from a different source.

Test item	Crossing ability + compliance + elastic recoil + longitudinal strength			
Brand	Abbott	Boston Scientific	Microport	HELIOS
Type	XIENCE Xpedition	SYNERGY	FIREHAWK	HELIOS
Size	3.0 ∗ 38	3.0 ∗ 38	3.0 ∗ 38	3.0 ∗ 38
Sample size	3	3	3	3

## Data Availability

The data used to support the findings of this study are included within the article.

## Abbreviations

PCI: Percutaneous coronary intervention
BMS: Bare metal stents
ISR: In-stent restenosis
DES: Drug-eluting stent
MACE: Major adverse cardiac events
CAD: Coronary artery disease.
